# Supplementary material for: Effector-Memory B-Lymphocytes and Follicular Helper T-Lymphocytes as Central Players in the Immune Response in Vaccinated and Nonvaccinated Populations against SARS-CoV-2
Source: Vaccines (Basel). 2022 Oct 20;10(10):1761. doi: 10.3390/vaccines10101761 (PMC9607383; doi:10.3390/vaccines10101761)
Supplement: Supplementary file 1 [file vaccines-10-01761-s001.zip › Supplementary figures.pdf]

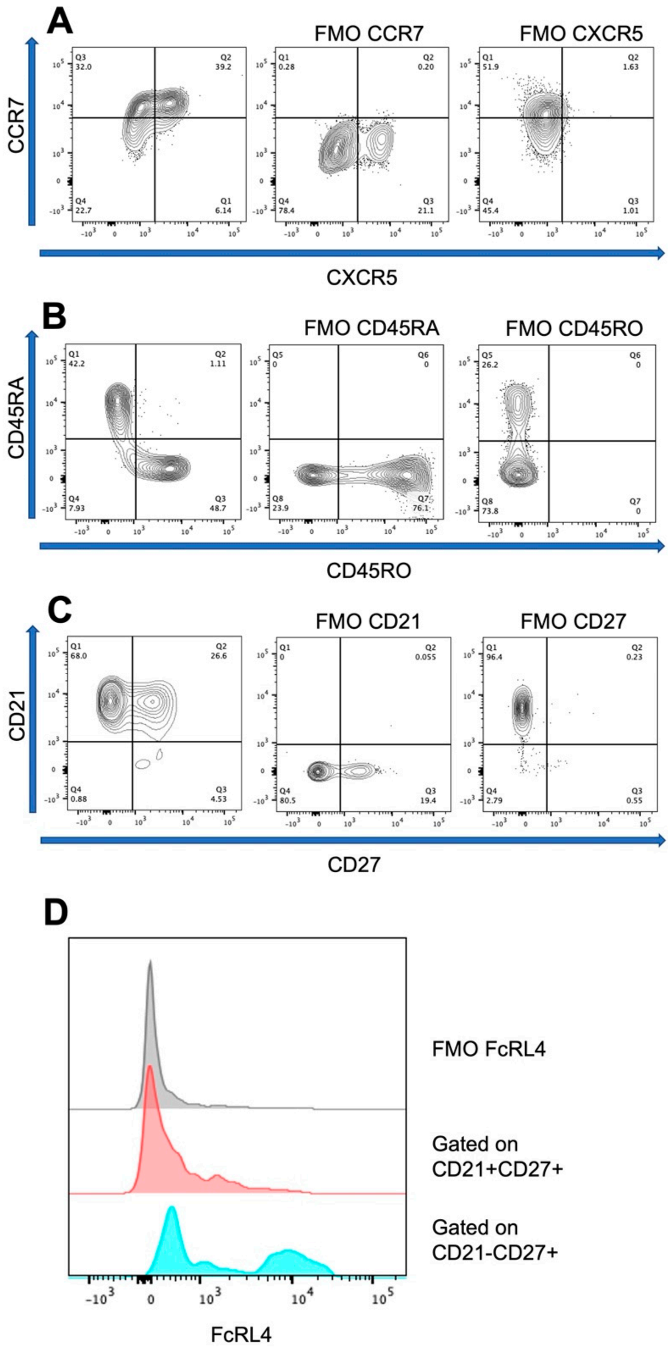

Figure S1: FMO controls. A. CCR7 vs CXCR5 dot plot from CD3+CD4+ lymphocytes. FMO for CCR7 and CXCR5 are shown. B. CD45RA vs CD45RO dot plot from CD3+CD4+ lymphocytes. FMO control for CD45RA and CD45RO are shown. C. CD21 vs CD27 dot plot from CD19+ lymphocytes. FMO control for CD21 and CD27. D. FcRL4 histogram gated on CD21+CD27+ and gated on CD21-CD27+ lymphocytes. FMO FcRL4 is shown.

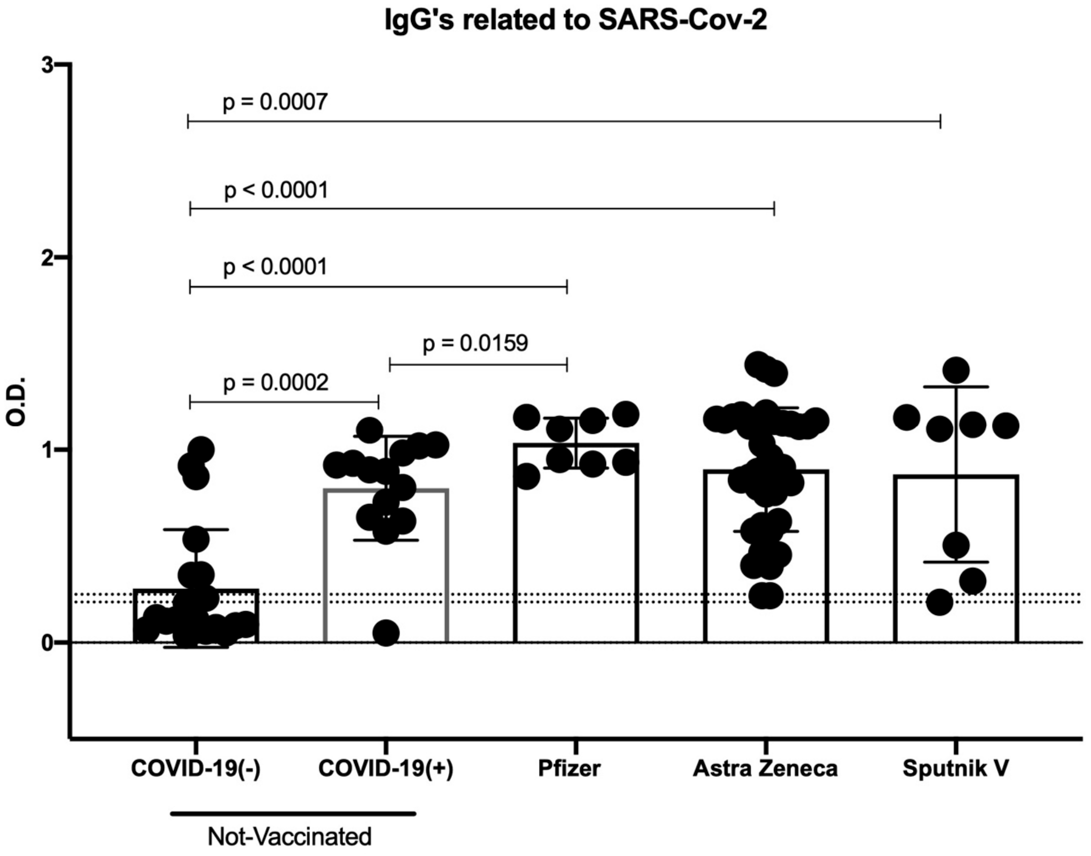

Figure S2: OD of IgGs related to SARS-CoV-2 against RBD domain of S protein induced by distinct vaccine platforms. Samples from non-vaccinated subject with and without history of COVID-19 (n = 14, and n = 20, respectively), and samples from subjects vaccinated with Pfizer (n = 8), vaccinated with Astra Zeneca (n = 38), and vaccinated with Sputnik V (n = 8). Cut-off values are indicated by lines. The mean ± standard deviation of the mean (SD) is shown.
